# Half-Yearly Report of Cases in Midwifery, Which Have Occurred in the Northern District of the London and Southwark Midwifery Institution

**Published:** 1831-02

**Authors:** C. Waller

**Affiliations:** Consulting Accoucheur to the above Institution, and Lecturer on Midwifery at the Medical School, 58, Aldersgate Street.


### MIDWIFERY.

*Half-yearly Report of Cases in Midwifery, which have occurred in the Northern District of the London and Southwark Midwifery Institution.* By C. Waller, Esq. Consulting Accoucheur to the above Institution, and Lecturer on Midwifery at the Medical School, 58, Aldersgate street.

| 1830. | *No. of Women delivered.* | *Sex of Children.* | | *Born Alive.* | *Still-born.* | *Presentation.* |
|---|---|---|---|---|---|---|
| | | *Males.* | *Females.* | | | |
| July .... | 31 | 17 | 14 | 30 | 1 | 30 Natural<br>1 Forehead |
| August... | 23 | 9* | 15 | 22 | 2 | 22 Natural<br>1 Premature<br>1 Face to Pubis |
| September | 27 | 16 | 11 | 27 | 0 | Natural |
| October. . | 39 | 21 | 18 | 36 | 3 | 37 Natural<br>2 Breech |
| November | 54 | 28 | 26 | 53 | 1 | Natural |
| December | 34 | 16† | 23 | 37 | 2 | 32 Natural<br>3 Breech<br>2 Feet<br>1 Hand & Foot<br>1 Face to Pubis |
| Total.... | 208 | 107 | 107 | 205 | 9 | |

Remarks. The present half-year has proved a much more favorable one for the patients of our institution, than the preceding; that formidable disease, puerperal fever, having nearly disappeared, two cases only having occurred, one of which proved fatal. This poor woman resided out of the district, but the case was kindly undertaken by a medical man in her neighbourhood, and consequently I did not see her.

In the second instance, the female had been delivered after a very natural labour, and appeared doing well until the third day, when she was seized with a rigor, followed by acute pain in the head, flushing of the face, and pain in the uterine region, considerably increased on pressure. I saw her about fourteen hours after the chill, and found her in the state just described; the pulse 150 in the minute, soft, and compressible; tongue moist; lacteal and lochial discharges not much affected. I ordered twenty leeches to be applied to the abdomen, and the following pills to be given immediately: ℞. Pulv. Opii gr. iv., Hydr. Submur. gr. v. fiat pil. ij. When seen a

* One case of twins.

† Three cases of twins, and one of triplets.

few hours afterwards, she expressed herself much relieved; the opium had rendered her giddy, but she had but slight pain. Ordered a bread and water poultice to be applied to the abdomen, and calomel with opium, in smaller doses, to be continued through the night. The medicine produced such a degree of purging, that we were obliged to discontinue it on the following morning, and to exhibit an opiate mixture. This state of bowels, with vomiting, teazed her for several days, but at length she recovered, though she was long in regaining her strength, and it was a considerable time before the pulse fell to its natural standard.

Several other cases of interest have occurred, but want of time prevented my taking sufficiently accurate notes to warrant their publication.

One case of acute Hysteritis has taken place, which destroyed the patient, (or, I should say, in which the patient's life was sacrificed to the want of good nursing:) she was seen early, and treated actively, and, although the disease at times appeared to be subsiding, yet it returned, and she died on the twelfth day after delivery. She was under the care of Mr. Bellis, a very intelligent man, and one of the senior pupils of the Aldersgate-street School, who saw her a few hours after the chill: she was then complaining of headach, and great pain in the abdomen; she had a hardish pulse of 120, and a white tongue, and, in fact, general symptoms of a febrile and inflammatory character. It should be observed, that the abdominal pain was confined to the uterus itself, the lochial exudation from which was nearly suspended, as was the lacteal secretion. A detailed account of the treatment would here be useless, as it was rendered nugatory by the dietetic plan adopted by the friends of this unfortunate woman: suffice it to say, that, within thirty-six hours after the chill, she had been bled four times from the arm, twenty leeches had been applied to the abdomen, and the mouth slightly affected by mercury; a plan hitherto almost invariably successful in cases of this kind: in truth, I never recollect being foiled in a similar instance, where this plan had been fully and fairly tried. At the time, however, we were employing these actively antiphlogistic measures, the attendants were plying the patient with gin, porter, and animal food, which, I apprehend, clearly accounts for the unsuccessful result. The dissection was interesting, as exhibiting a striking difference in the appearances after death between inflammation of the acute character, and that which occurs in the true and genuine puerperal fever. There was effusion to a considerable extent within the peritoneal cavity, and the different convolutions of the bowels generally adhering to each other. The omentum was also firmly bound down. This adhesion was very different in its nature to that agglutination which is sometimes seen in fatal cases of puerperal fever: it arises, I believe, in this latter instance, simply from the curdy matter so copiously effused in this disease, being deposited between the intestinal folds, and not from any organized adhesions; for, in some of the worst cases I ever saw, the intestines were floating as loosely as natural. The lumps of adhesive matter which were mixed with the effusion before noticed, were of a firmer consistence than that seen in puerperal fever. The fallopian tubes were filled with pus, and the blood-vessels of the ovaries somewhat turgid; the uterus itself appeared to have become free from disease; the extension of the inflammation along the peritoneal membrane evidently being the cause of death, and I should, without hesitation, say that this effect was produced in consequence of the injudicious use of food and stimulants.

Several cases of Hemorrhage have occurred within the half year, some of them pretty severe: they have, however, all recovered rapidly, under the ordinary method of treatment.

Four cases of twins have been reported, three of which, and one case of triplets, happened within the short space of ten days. The number of twin and triplet cases occurring in this neighbourhood, within the last two or three months, has been very remarkable, exceeding by far any thing of the kind within my recollection. In the instance of triplets in our own institution, there were two boys and a girl, all of whom, with the mother, are likely to do well. The two boys were born before the girl; the first presented the breech, the second the feet; and lastly the girl was expelled, and this also was a breech presentation. The poor woman, as might be expected, is feeling low and weak; but, if she can procure sufficient nourishment, there appears to be every chance of the children doing well. A number of benevolent individuals having come forward to assist her, she has not at present suffered. The children were born on the 14th of December.

93, *Bartholomew Close; January,* 1831.

---

